# Keishikaryukotsuboreito Potentiates NGF-Induced Neurite Outgrowth in PC12 Cells

**DOI:** 10.3390/life16030423

**Published:** 2026-03-05

**Authors:** Kazuki Terada, Yukari Matsushima, Makoto Hosoyamada

**Affiliations:** 1 Laboratory of Human Physiology and Pathology, Faculty of Pharmaceutical Sciences, Teikyo University, Tokyo 173-8605, Japan; 2 Ginza Research Center, Mirailab Bioscience Inc., Tokyo 104-0061, Japan; y.terada@mirailab-bio.com

**Keywords:** Kampo medicine, nerve growth factor, neurite outgrowth, Keishikaryukotsuboreito, Keishito

## Abstract

Keishikaryukotsuboreito (KKT) is a Kampo formula prescribed for neuropsychiatric symptoms, whereas Keishito (KT), despite including most of the constituent herbs, is not indicated for such conditions, suggesting distinct biological actions. We examined whether KT and KKT modulate nerve growth factor (NGF)-induced neurite outgrowth in PC12 cells. Cells were stimulated with NGF in the presence or absence of KT or KKT, and neurite extension was quantified. The involvement of NGF receptor signaling was assessed using the Trk inhibitor K-252a. KKT, but not KT, enhanced NGF-induced neurite outgrowth in a concentration-dependent manner without affecting basal morphology. Pharmacological analysis showed that KKT increased the maximal NGF-induced neurite response (E_max_) without altering NGF potency (EC_50_). K-252a completely abolished NGF-induced neurite extension and KKT-mediated enhancement, indicating that the effect was entirely dependent on NGF–TrkA signaling. These findings demonstrate that KKT selectively augments NGF-elicited neuronal differentiation and suggest translational relevance as a neurotrophic strategy.

## 1. Introduction

Nerve growth factor (NGF) promotes neurite outgrowth and neuronal differentiation through activation of its high-affinity receptor TrkA [1]. Because of these properties, NGF has attracted interest as a potential therapeutic agent for neurodegenerative diseases, including Alzheimer’s disease [2]. However, clinical application of NGF is limited due to poor blood–brain barrier permeability and associated delivery challenges, which may result in adverse effects [3]. Traditional herbal medicines have long been used in East Asia to treat neurological and psychiatric conditions, and several formulations have been reported to modulate neurotrophic signaling pathways [4]. Keishikaryukotsuboreito (KKT) is clinically prescribed for symptoms such as anxiety, insomnia, palpitations, and nervous irritability [5]. In contrast, Keishito (KT), despite sharing multiple herbal components with KKT, is primarily used in the early stages of the common cold and is not typically indicated for neuropsychiatric disorders [6]. These distinct clinical indications suggest that the two formulas may exert different effects on neuronal function, despite their compositional similarity. Given that neuropsychiatric symptoms often involve alterations in neuronal plasticity or excitability, we hypothesized that KKT may modulate NGF-induced neuronal differentiation at the cellular level.

PC12 cells, which express functional TrkA receptors and exhibit reproducible neurite extension upon NGF stimulation, are widely used as a model of NGF-dependent neuronal differentiation. The purpose of this study was to compare the effects of KKT and KT on NGF-induced neurite outgrowth and to determine whether any observed effects depend on TrkA signaling.

## 2. Materials and Methods

### 2.1. Reagents

NGF (murine NGF 2.5S, derived from mouse submaxillary glands; Alomone Labs Ltd., Jerusalem, Israel) and K-252a (COSMO BIO, Tokyo, Japan) were used in this study. The crude herbs used to prepare the Kampo decoctions were obtained from Uchida Wakanyaku Co., Ltd. (Tokyo, Japan), for Cinnamomi Cortex, Paeoniae Radix, Zizyphi Fructus, and Glycyrrhizae Radix, as well as from Tsumura & Co. (Tokyo, Japan) for Zingiberis Rhizoma, Fossilia Ossis Mastodi, and Ostreae Testa. HPLC-grade acetonitrile and ammonium acetate were purchased from FUJIFILM Wako Pure Chemical Corporation (Osaka, Japan).

### 2.2. Preparation of Kampo Formulations

**Figure 1:**
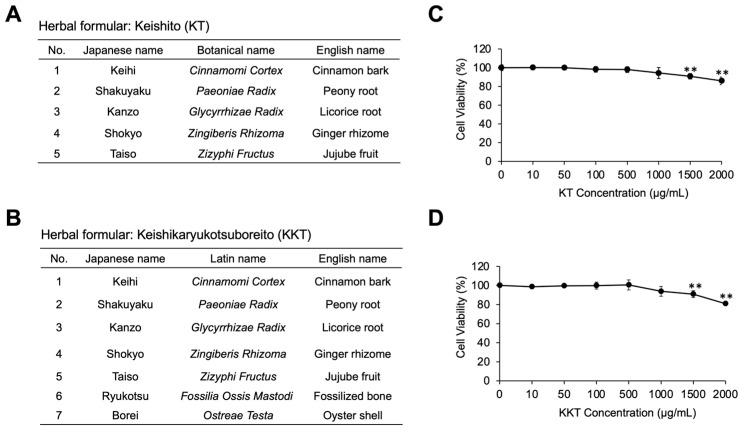
Crude herbal compositions of Keishito (KT) and Keishikaryukotsuboreito (KKT) and effects on PC12 cell viability. (**A**,**B**) Constituent crude herbs of KT and KKT. (**C**,**D**) PC12 cells were treated with KT or KKT (0–2000 µg/mL) for 24 h, and viability was measured using CCK-8 assay. Data are presented as mean ± SD (*n* = 3). ** *p* < 0.01 vs. control (0 µg/mL).

The constituent crude herbs of KT and KKT are shown in Figure 1A,B, respectively. KT and KKT were prepared according to the ingredient compositions described by Oguri et al. [5] and Yoo et al. [6], respectively. KT was prepared by mixing Cinnamomi Cortex (4.0 g), Paeoniae Radix (4.0 g), Zizyphi Fructus (4.0 g), Glycyrrhizae Radix (2.0 g), and Zingiberis Rhizoma (1.5 g). KKT was prepared by adding Fossilia Ossis Mastodi (3.0 g) and Ostreae Testa (3.0 g) to the KT formulation. Each mixture was decocted in 600 mL of purified water for 1 h in a hot water bath, cooled, and filtered. The filtrates were subsequently concentrated using a rotary evaporator(N-1300, Tokyo Rikakikai Co., Ltd., Tokyo, Japan) and dried in a vacuum dryer to obtain powdered samples.

### 2.3. Cell Culture

PC12 cells were obtained from the RIKEN Cell Bank (Ibaraki, Japan) and maintained at 37 °C in a humidified incubator with 5% CO_2_. Cells were grown in DMEM/F-12 medium containing 10% fetal bovine serum and 1% penicillin–streptomycin (Gibco, Life Technologies, Carlsbad, CA, USA).

### 2.4. Cell Viability Assay

The cytotoxicity of KKT and KT was assessed using a Cell Counting Kit-8 assay (CCK-8; Dojindo, Kumamoto, Japan). PC12 cells were seeded in 96-well plates (1 × 10^4^ cells/well), exposed to the indicated concentrations for 24 h, and incubated with CCK-8 reagent for 4 h, before absorbance at 450 nm was measured using a DTX 880 microplate reader (Beckman Coulter, Brea, CA, USA).

### 2.5. HPLC Analysis

The sample was filtered through a 0.45 μm membrane filter and analyzed by HPLC under the following conditions: column, CAPCELL PAK C18 (Osaka Soda Co., Ltd.; 5 μm, 4.0 mm i.d. × 25 cm, Osaka, Japan); mobile phase, CH_3_CN:CH_3_COONH_4_ (linear gradient, 1:9→7:3); flow rate, 0.7 mL/min; column temperature, 40 °C; injection volume, 20 μL; detection, UV absorbance at 254 and 280 nm. Each sample (100 mg) was mixed with 10 mL of water, allowed to stand at room temperature, and then filtered prior to injection. Reference standards of paeoniflorin, liquiritin, cinnamic acid, and glycyrrhizic acid were purchased from FUJIFILM Wako Pure Chemical Corporation (Osaka, Japan).

### 2.6. Measurement of Neurite Length

Total neurite length was used as an index of NGF-induced neurite outgrowth, as previously described by Terada et al. [7]. PC12 cells were treated for 24 h with NGF (50 ng/mL) in DMEM/F-12 containing 5% FBS, either alone or together with KKT or KT. After incubation, images were acquired from five randomly selected fields per dish using a phase-contrast microscope (ECLIPSE TS100; Nikon, Tokyo, Japan) equipped with a Digital Sight DS-L2 camera system. Neurite length in each image was quantified using ImageJ software (version 1.53, National Institutes of Health, Bethesda, MD, USA), and the mean value from the five fields was defined as the neurite outgrowth for each dish (*n* = 3). NGF concentration–response curves were analyzed by nonlinear regression with a four-parameter logistic model using GraphPad Prism (version 8.4.3; GraphPad Software, San Diego, CA, USA). Emax and EC50 values, together with their 95% confidence intervals (CIs), were obtained from the fitted curves.

### 2.7. Pharmacological Inhibition of the TrkA in PC12 Cells

To examine the involvement of TrkA signaling, PC12 cells were pretreated with the Trk inhibitor K-252a (100 nM) for 30 min prior to stimulation with NGF in the presence or absence of KKT or KT. Cells were then incubated for 24 h, and neurite outgrowth was quantified as described above.

### 2.8. Statistical Analysis

Quantitative data, expressed as mean ± SD, were evaluated using analysis of variance (ANOVA), followed by Tukey’s post hoc test. The level of statistical significance was set at *p* < 0.05.

## 3. Results

### 3.1. Influence of KT and KKT on PC12 Cell Viability

KT and KKT were evaluated for cytotoxicity across a broad concentration range (10–2000 µg/mL). Both formulations maintained cell viability comparable to that of untreated controls at concentration levels up to 1000 µg/mL. However, a significant reduction in viability was observed for both KT and KKT at higher concentrations (1500 and 2000 µg/mL) (Figure 1A,B), indicating the onset of cytotoxicity above this threshold. Based on these findings, concentrations ≤ 1000 µg/mL were considered non-toxic and were used for subsequent neurite outgrowth experiments.

### 3.2. Composition and Component Analysis of KT and KKT

**Figure 2:**
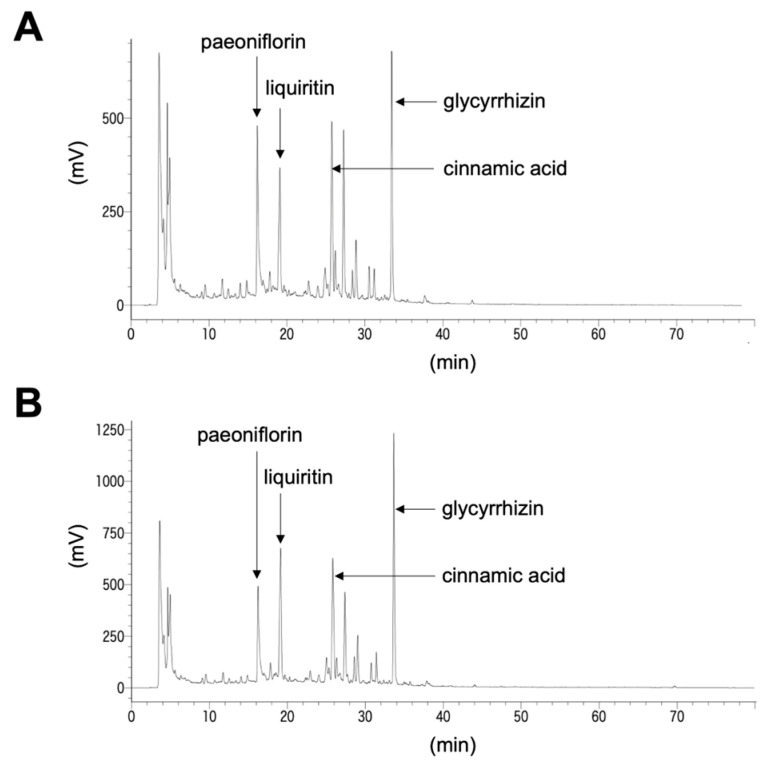
Representative HPLC chromatograms of (**A**) KT and (**B**) KKT. Major constituents were identified by comparison with reference standards, including paeoniflorin, liquiritin, cinnamic acid, and glycyrrhizic acid.

The herbal compositions of KT and KKT are shown in Figure 1. To further characterize the chemical components of these formulations, HPLC analysis was performed on KT and KKT. Representative chromatograms are shown in Figure 2. Several major constituents were identified by comparing their retention times and UV absorbance profiles with those of reference standards analyzed separately under identical chromatographic conditions, including paeoniflorin derived from Paeoniae Radix (Shakuyaku), liquiritin and glycyrrhizic acid derived from Glycyrrhizae Radix (Kanzo), and cinnamic acid derived from Cinnamomi Cortex (Keihi). These components were detected in both KT and KKT (Figure 2).

### 3.3. KKT Enhances NGF-Induced Neurite Outgrowth

**Figure 3:**
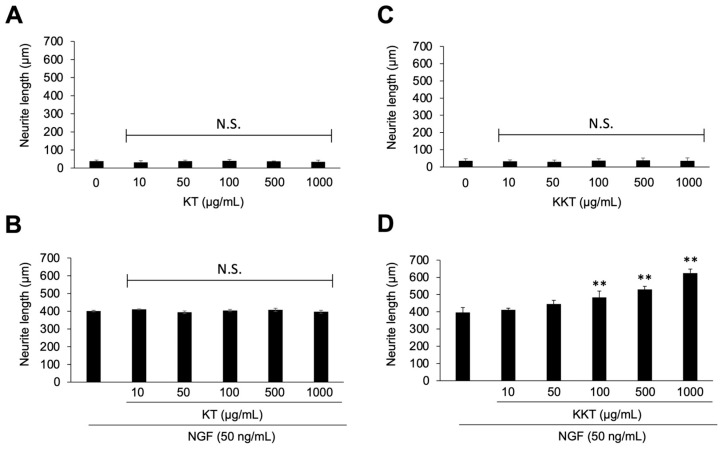
Effects of KT and KKT on NGF-induced neurite outgrowth in PC12 cells. PC12 cells were treated for 24 h with KT or KKT (0–1000 µg/mL) in the (**A**,**C**) absence or (**B**,**D**) presence of NGF (50 ng/mL). Total neurite length quantified from five random fields per dish. Data are presented as mean ± SD (*n* = 3). Scale bars: 50 µm. ** *p* < 0.01 vs. respective controls (0 µg/mL or NGF alone); N.S., not significant.

Treatment with KT did not modify neurite morphology under either basal conditions or NGF stimulation (Figure 3A,B). KKT alone did not induce neurite outgrowth; however, in the presence of NGF, it significantly increased NGF-induced neurite outgrowth in a concentration-dependent manner (Figure 3C,D). These results show that KT was inactive in this experimental setting, whereas KKT selectively enhanced NGF-induced neurite outgrowth.

### 3.4. TrkA-Dependent Potentiation of NGF-Induced Neurite Outgrowth by KKT

**Figure 4:**
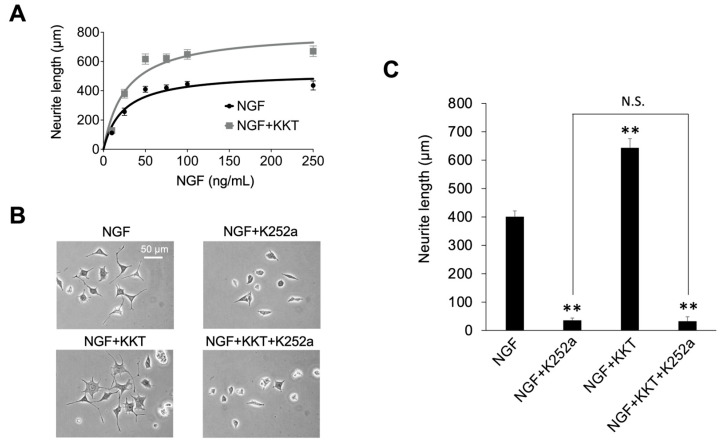
KKT increases the maximal NGF-induced neurite outgrowth, and this effect is blocked by K-252a. (**A**) NGF concentration–response curves for neurite outgrowth in the absence or presence of KKT. (**B**) Representative images of neurite outgrowth with NGF, KKT, and/or K-252a (100 nM). Scale bars: 50 µm. (**C**) Quantification of neurite outgrowth. Data are presented as mean ± SD (*n* = 3). ** *p* < 0.01 vs. NGF; N.S., not significant.

KKT increased the maximal neurite outgrowth response (E_max_) to NGF without altering EC_50_ (Figure 4A). The E_max_ value for NGF alone was 526.6 μm (95% CI: 476.4–584.1), whereas co-treatment with KKT increased the E_max_ to 806.4 μm (95% CI: 721.9–905.0) (Figure 4A). In contrast, the EC_50_ values were not significantly different between conditions—NGF alone showed an EC_50_ of 23.4 ng/mL (95% CI: 17.1–34.1), and NGF + KKT showed an EC_50_ of 25.4 ng/mL (95% CI: 16.9–37.2) (Figure 4A). These data indicate that KKT enhances the E_max_ of NGF-induced neurite outgrowth without affecting EC_50_. To determine whether this potentiation required NGF receptor signaling, the effect of the TrkA inhibitor K-252a was examined. Co-treatment with K-252a completely abolished the enhancement mediated by KKT and reduced neurite length to the level observed with NGF and K-252a alone (Figure 4B,C).

Thus, the potentiating effect of KKT on NGF-induced neurite outgrowth requires TrkA-mediated NGF signaling.

## 4. Discussion

Although KT and KKT share many of their constituent herbs, their markedly different clinical indications suggest that subtle compositional differences or herb–herb interactions may lead to distinct biological effects. This distinctiveness prompted an investigation of whether the two formulations differed in their effects on NGF-related neuronal differentiation.

The present results demonstrate that KKT significantly potentiates NGF-evoked neurite extension in PC12 cells (Figure 3). Importantly, KKT increased the E_max_ of NGF-induced neurite extension without altering the EC_50_ (Figure 4A). This divergence between efficacy and sensitivity suggests that the affinity or responsiveness of TrkA signaling is not enhanced by KKT; instead, the downstream capacity of the NGF-induced differentiation program is augmented. Such a selective enhancement of E_max_ is consistent with facilitation of cytoskeletal remodeling or signal amplification mechanisms that operate after TrkA activation.

Further supporting this interpretation, K252a completely abolished the NGF-induced neurite outgrowth and eliminated the additional enhancement produced by KKT (Figure 4C). This dual loss of activity indicates that the potentiating effect of KKT is mediated through NGF–TrkA signaling. Together, these findings suggest that KKT strengthens NGF-elicited differentiation signals, rather than initiating neurite outgrowth on its own. To our knowledge, no previous study has reported that KKT potentiates NGF–TrkA-dependent neurite outgrowth. Importantly, this study directly compares KT and KKT at the cellular level and shows that subtle compositional differences result in distinct NGF-dependent neurotrophic activity.

Previous research has suggested that certain Kampo formulas exert modulatory effects on neurotrophic signaling pathways relevant to neuronal differentiation. These findings imply that some traditional formulations with neuropsychiatric indications may influence NGF-related cellular responses, although the extent and mechanisms of such actions appear to vary among individual formulas. Among the Kampo medicines, Yokukansan (YKS), a formula used clinically for neurosis and insomnia [8,9], has also been reported to enhance NGF-induced neurite outgrowth [7]. Although YKS and KKT share Paeoniae Radix and Glycyrrhizae Radix, these herbs are also present in KT and showed no activity in our analysis. Thus, these common components are unlikely to account for the KKT-specific effects. To further explore this, the ability of extracts from *Fossilia Ossis Mastodi* (ryukotsu) or *Ostreae Testa* (borei), the major ingredients distinguishing KKT from KT, to reproduce KKT’s effects was evaluated. However, none of the extracts exhibited KKT-like activity.

These findings suggested that the neurotrophic effects of KKT cannot be attributed to a single constituent herb. Herbal formulations frequently exert biological activities arising from multicomponent interactions, and synergistic effects have been reported for several Kampo medicines [10,11]. Therefore, the inability of isolated components to mimic KKT supports the possibility that its NGF-potentiating action results from higher-order interactions among shared and unique constituents.

## 5. Conclusions

This study demonstrates that Keishikaryukotsuboreito selectively potentiates NGF-induced neurite outgrowth in PC12 cells, whereas Keishito has no effect. KKT increased the maximal NGF response without altering NGF potency, and Trk inhibition completely abolished both NGF-induced neurite outgrowth and the KKT-mediated enhancement, indicating that the effect is entirely dependent on NGF–TrkA signaling. These findings suggest that the neurotrophic action of KKT arises from multicomponent interactions, rather than from a single herbal constituent, and highlight its potential relevance as an approach for enhancing NGF responsiveness. Given the critical role of NGF–TrkA signaling in neuronal survival and plasticity, the present findings may have implications for therapeutic strategies targeting neurodegenerative or neuropsychiatric conditions associated with impaired neurotrophic signaling.

## Data Availability

The original contributions presented in this study are included in the article. Further inquiries can be directed to the corresponding authors.

## Abbreviations

The following abbreviations are used in this manuscript:

KKT	Keishikaryukotsuboreito
KT	Keishito
NGF	Nerve growth factor
TrkA	Tropomyosin receptor kinase A
YKS	Yokukansan
